# The Association between Early Opioids Prescribing and the Length of Disability in Acute Lower Back Pain: A Systematic Review and Narrative Synthesis

**DOI:** 10.3390/ijerph191912114

**Published:** 2022-09-25

**Authors:** Ayman R. Ibrahim, Mohamed E. Elgamal, Moaz O. Moursi, Bara A. Shraim, Muath A. Shraim, Mujahed Shraim, Basem Al-Omari

**Affiliations:** 1 College of Medicine, QU Health, Qatar University, Doha P.O. Box 2713, Qatar; 2 Department of Internal Medicine, Hamad General Hospital, Hamad Medical Corporation, Doha P.O. Box 3050, Qatar; 3 Department of Plastic and Reconstruction Surgery, Hamad General Hospital, Hamad Medical Corporation, Doha P.O. Box 3050, Qatar; 4 NHMRC Centre of Clinical Research Excellence in Spinal Pain, Injury & Health, School of Health & Rehabilitation Sciences, The University of Queensland, St Lucia, QLD 4072, Australia; 5 Department of Public Health, College of Health Sciences, Qatar University, QU Health, Doha P.O. Box 2713, Qatar; 6 College of Medicine and Health Sciences, Khalifa University of Science and Technology, Abu Dhabi P.O. Box 127788, United Arab Emirates; 7 KU Research and Data Intelligence Support Center (RDISC) AW 8474000331, Khalifa University of Science and Technology, Abu Dhabi P.O. Box 127788, United Arab Emirates

**Keywords:** opioids, opiates, low back pain, return to work, sick leave, length of disability, systematic review

## Abstract

Background: There is conflicting evidence with respect to whether early opioid prescribing (EOP) within the first two weeks of acute Low Back Pain (LBP) onset is associated with the length of disability (LOD). The aim of this systematic review was to examine the relationship between EOP and LOD in individuals with acute LBP. Methods: A systematic search of Medline, EMBASE, and CINAHL was conducted. The Newcastle–Ottawa scale was used to assess the methodological quality of included studies. A narrative synthesis of findings was used owing to between-study heterogeneity. Results: Six cohort studies using workers’ compensation administrative data on 178,130 adults with LBP were included. Most studies were of good methodological quality. One study reported that LBP cases with EOP had higher LOD by 4 days than cases without EOP. Two studies reported that each 100 mg morphine equivalent amount (MEA) was associated with an increase in mean LOD by 0.4 day (95% confidence interval (CI): 0.3, 0.5) and 0.4 day (95% CI: 0.3, 0.4). One study showed that LBP cases with EOP had a higher hazard of continuation of time loss benefits by 1.94 (95% CI 1.86, 2.02). One study reported a dose–response relationship between MEA of EOP and LOD ranging between 5.2 days (95% CI 14.6, 25.0) for 1–140 mg MEA and 69.1 (95% CI 49.3, 89.0) for 450+ mg MEA. One study reported that LBP cases with EOP had a higher mean LOD by 3.8 days, but there was no statistically significant relationship between EOP and LOD (Hazard ratio 1.02; 95% CI 0.91, 1.13). Conclusions: The use of early opioid in the management of acute uncomplicated LBP is associated with prolonged disability duration. Further research on factors influencing inadequate adherence to evidence-based guidelines and optimal strategies to modify such factors may improve disability outcomes among patients presenting with acute LBP.

## 1. Introduction

Lower back pain (LBP) is a global public health problem. Most people of all age groups experience at least one episode of LBP during their lifetime [1,2]. In 2019, the global age-standardized point prevalence and incidence of LBP were 7% (95% confidence interval (CI) 6.2, 7.9) and 2.7% (95% CI 2.4, 3.1), respectively [3]. In the Global Burden of Disease study conducted in 2019, LBP was the third leading cause of years lived with disabilities (YLDs), accounting for 7.4% (95% CI 6.2, 8.7) of the global YLDs [4]. Most acute episodes of LBP recover spontaneously within a few weeks, but about one-third and one-quarter of people with LBP experience recurrent and chronic LBP, respectively [5,6]. Persistent LBP is associated with adverse long-term outcomes on the individual (e.g., depression, anxiety, sleep disorders, job dissatisfaction, prolonged length of disability, and negative body image) [7,8] and results in very high costs to the healthcare system associated with medical care and work disability [3,9,10,11].

Several factors were found to be associated with persistent and disabling LBP, including LBP-related factors (previous episodes of LBP, LBP intensity, and presence of leg pain), lifestyle factors (obesity, smoking, and low levels of physical activity), psychological factors (depression, catastrophizing, and fear avoidance beliefs), and occupational and socioeconomic factors (physical workloads, low education level, workers’ compensation (WC) policies, and dissatisfaction with work) [1,12,13,14,15]. In addition, healthcare factors such as early MRI scanning within four weeks of LBP onset and, of particular interest, early opioid prescribing (EOP) within two weeks of LBP onset were identified as strong predictors of an increased length of disability (LOD) [16,17]. In addition, visceral referred pain, which is a vague pain originating from visceral organs that is felt at another body location, is a commonly overlooked cause of musculoskeletal pain [18,19]. It can be explained, at least in part, by the sensitization of peripheral nerves that occurs in acute and chronic systemic inflammatory processes [20]. For example, gastrointestinal complaints, such as diarrhea, were found to be associated with LBP [21,22].

EOP within the first two weeks of LBP onset may be crucial in enabling individuals with acute LBP an earlier return to daily activities. However, adverse side effects such as drowsiness, cognitive impairment [23], addiction, and overdosing may place the individual at risk and delay recovery [24,25]. Clinical guidelines emphasize the avoidance of opioids in the management of acute LBP, with a focus on reassurance, staying active, and the use of non-steroidal anti-inflammatory drugs and acetaminophen if needed, with opioids reserved only for severe and unresponsive cases and only for several days [26,27,28]. Despite these recommendations, around 60% and 28% of cases presenting with LBP in ED and primary care are prescribed EOP, respectively [15,29]. EOP in acute LBP is associated with an increased LOD at two years of follow-up [17], a higher risk for surgery [17,30], and increased healthcare utilization (e.g., physiotherapy, imaging, hospital admission, and surgery) [30,31]. However, one study by Lee and colleagues demonstrated conflicting findings in which no statistically significant association was found between EOP and LOD in acute LBP [30]. To clarify and consolidate these conflicting findings, this study aims to undertake a systematic approach to review the literature, investigating the relationship between EOP and LOD in individuals with acute uncomplicated LBP.

## 2. Materials and Methods

The protocol for this review was registered with the International Prospective Register of Systematic Reviews (PROSPERO) under registration number CRD42021260723 (available from: https://www.crd.york.ac.uk/prospero/display_record.php?RecordID=260723, accessed on the 26 August 2022).

### 2.1. Search Strategy

The report of this systematic review was guided by the Preferred Reporting Items for Systematic Reviews and Meta-Analysis (PRISMA) statement [32]. A comprehensive search utilizing Medline, EMBASE, and CINAHL databases from their inception until 14 March 2022 was conducted. Controlled vocabularies and free-text terms on opioids, LBP, and work disability were used (Supplemental File S1). In addition, experts in the field were contacted, reference lists of all relevant papers were screened, and citations of included papers were traced using the Web of Science Citation Index. No restrictions on the language of publication or study design were applied.

### 2.2. Criteria for Considering Studies for the Review

#### 2.2.1. Inclusion Criteria

All epidemiologic study designs examining the association between EOP and LOD in adults presenting with acute uncomplicated LBP were considered for inclusion. EOP was defined as prescribing opioid medications for LBP within the first 15 days of seeking medical treatment for acute LBP [17]. Therefore, studies including adults (aged 18 years and older) presenting with an acute, occupational, or non-specific LBP were eligible for inclusion. The outcome measure was the measure of association between EOP and LOD defined as the number of work disability days (absence from work) due to the current episode of LBP [14,33].

#### 2.2.2. Exclusion Criteria

Studies were excluded if they included patients with chronic or complicated LBP, such as multiple and severe injuries, infection, cancer, or autoimmune disease.

### 2.3. Study Selection Process

**Figure 1:**
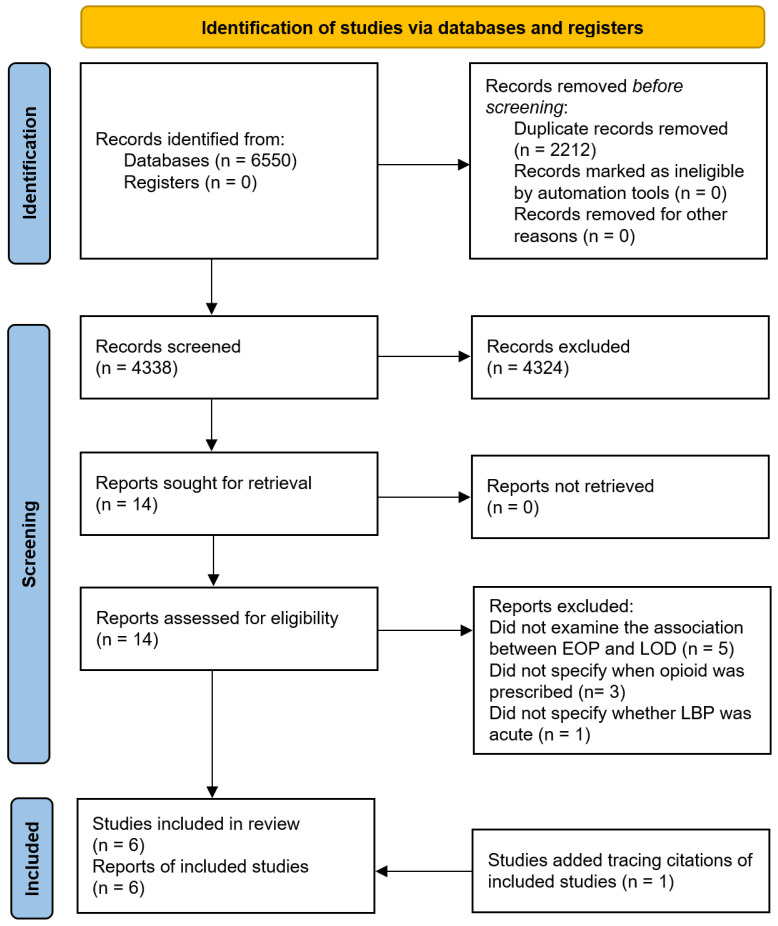
The PRISMA flow diagram of studies in the review.

All identified papers were independently reviewed by two reviewers and any disagreements were resolved by a third independent adjudicator. Retrieved records were imported to the Covidence web-based application, and duplicates were deleted. In the first stage, titles and abstracts of the remaining studies were screened against the inclusion and exclusion criteria, and irrelevant studies were excluded. In the second stage, full-texts of all potentially relevant studies, or when a decision could not be made based on the titles and abstracts, were retrieved and reviewed against the inclusion and exclusion criteria. Reasons for study exclusions at the full-text review stage are reported in the PRISMA flow diagram (Figure 1). The study selection process was performed independently by two reviewers, and any disagreements were resolved through consensus-based discussion or by involving a third reviewer.

### 2.4. Quality Assessment

A quality assessment of included studies was conducted independently by two reviewers using the Newcastle–Ottawa scale (NOS) for cohort studies (Supplemental File S2) [34], and any disagreements were resolved by discussion or involving a third reviewer until a consensus was achieved. This tool assesses the quality of cohort studies using a “star system” for three main perspectives including selection of study cohorts, comparability of study cohorts, and the ascertainment of the outcome of interest. Each study can be given a maximum of eight 8 stars (4 stars for the process of cohorts’ selection, 2 stars for the comparability of the study cohorts, and 3 stars for ascertainment of the outcome). To summarize the methodological quality for each included study, the NOS was converted to one of the following methodological quality criteria recommended by the Agency for Healthcare Research and Quality: (a) good methodological quality (3 or 4 stars for selection of cohorts, 1 or 2 stars for comparability of cohorts, and 2 or 3 stars for ascertainment of the outcome); (b) fair methodological quality (2 stars for selection of cohorts, 1 or 2 stars for comparability of cohorts, and 2 or 3 stars ascertainment of the outcome); (c) poor methodological quality (0 or for selection of cohorts, 0 stars for comparability of cohorts, or 0 or 1 stars ascertainment of the outcome) [35].

### 2.5. Data Extraction

Data on the following items were extracted from each included study: aim, design, setting, source of data, study population, sample size, inclusion and exclusion criteria, follow-up duration, independent variables controlled for in multivariable analysis, and summary measures of association between EOP and LOD with corresponding CI. All necessary data were available in all included studies. Therefore, no authors were contacted. Two reviewers extracted the data from included studies independently, and disagreements were addressed by discussion or involvement of a third reviewer. One of the current review authors (MS) co-authored two of the included studies. Therefore, to avoid any potential reviewer bias, MS was not involved in data extraction or the risk of bias assessment for those two studies.

### 2.6. Data Analysis

We considered performing a meta-analysis. However, this was not feasible due to heterogeneity between included studies in measures of association between EOP and LOD. Therefore, a narrative synthesis was used.

## 3. Results

### 3.1. Study Selection

This systematic review search identified 6550 records. Of these, 2212 duplicates were removed, and 4324 records were excluded based on screening of titles and abstracts. After the full-text evaluation of the remaining 14 studies, nine studies did not meet the inclusion criteria and were excluded. Furthermore, one study was identified by tracing citations of included papers. A total of six studies were included in this systematic review. A flow diagram of the literature search and study selection process is shown in Figure 1.

### 3.2. Study Characteristics

Table 1 presents the characteristics of included studies. All included studies collected data from WC administrative databases and used a retrospective cohort design. Five studies were performed in the United States (US) [15,17,30,36,37], and one was performed in Canada [38]. Two of the included papers used the same sample to explore the association between various individual-level (including EOP), neighborhood, and state-level variables and LOD in patients with LBP [15,37]. However, each of the two studies addressed a different objective meeting the inclusion criteria of this review. The total number of individuals with LBP was 178,130 with a sample size ranging between 2887 and 59,656. The mean age of participants ranged from 37 to 41 years. All studies included more men than women with proportions ranging between 62% and 72% (Table 1). The studies involved cases with acute uncomplicated LBP that were identified using the International Classification of Diseases, Ninth Revision (ICD-9) codes [15,17,30,36,37], or the nature of injury and body part codes [38]. EOP was described as an opioid prescription within two days of the initial ED visit [30], a week of the first medical visit [36], or the first 15 days of the injury or filing a WC claim [15,17,37,38]. The LOD was described as the number of days lost from work estimated based on temporary total and temporary partial wage replacement payments truncated at one year [15,30,36,37,38] or two years [17].

### 3.3. Quality Assessment

Five studies demonstrated good methodological quality [15,17,30,37,38] and the remaining study [36] was of poor quality [36] (Table 2). Four studies scored 9 starts on the NOS in terms of selection, comparability, and outcome [15,17,30,37]. One study scored 8 out of 9 stars for not adjusting for LBP severity [38]. One study scored 7 out of 9 stars, with no stars given for the comparability domain for reporting only descriptive statistics describing the relationship between EOP and LOD [36].

### 3.4. The Association between EOP and LOD

All included studies aimed to investigate the association between EOP and LOD. Five studies used multivariable analyses while consistently adjusting for age and gender as potential confounders, whereas one study did not report adjusted analysis (Table 3). The follow-up period for all studies was one year except for one study, which had a follow-up duration of two years [17].

One study showed that LBP cases who were prescribed opioids within the first week of the first medical visit had a higher median LOD by four days than those who were not prescribed opioids at one year of follow-up [36]. Two studies reported that each 100 mg morphine equivalent amount (MEA) prescribed within the first two weeks of LBP onset was associated with an increase in the adjusted geometric mean of LOD by 0.4 day (95% CI: 0.3, 0.5) [37] and 0.4 day (95% CI: 0.3, 0.4) at one year of follow-up [15]. One study showed that cases who were prescribed opioids within the first two weeks of LBP onset had a higher hazard of continuation of time loss benefits by 1.94 (95% CI 1.86, 2.02) than cases who did not receive EOP at one year of follow-up [38]. One study reported that, compared to cases who were not prescribed opioids within the first two weeks of LBP onset, cases who received EOP had increased mean LOD days with an increasing MEA in a linear dose–response relationship over two years of follow-up: 5.2 days (95% CI −14.6, 25.0) for 1–140 mg MEA, 21.9 days (95% CI 3.2, 40.6) for 141–225 mg MEA, 43.8 days (95% CI 23.7, 63.9) for 226–450 mg MEA, and 69.1 (95% CI 49.3, 89.0) for 450+ mg MEA [17]. The last study reported that LBP cases who received opioid prescriptions within 2 days of the initial ED visit had a higher mean LOD by 3.8 days than those who were not prescribed opioids, but no statistically significant relationship was observed between EOP and LOD at one year of follow-up (hazard ratio 1.02; 95% CI: 0.91, 1.13) [30].

## 4. Discussion

This is the first study to systematically review the relationship between EOP in the first two weeks of LBP onset and the LOD in patients with acute uncomplicated LBP. Six studies with a total of 178,130 LBP cases were included in this review. The mean age of the participants was approximately 40 years with the majority being males (~70%). The samples included in the studies were representative of their respective population as they were collected from WC administrative data from the state of California [36], the entire US [15,17,30,37], and the WC Board of Alberta, Canada, which covers the majority of injured workers in that province [38].

Overall, the six studies showed that LBP cases prescribed early opioids had increased LOD duration than cases who were not prescribed early opioids at one and two years of follow-up. Only five studies presented adjusted associations between EOP and LOD [15,17,30,37,38]. Of these five studies, four studies found statistically significant associations between EOP and LOD [15,17,37,38], with one study showing a dose–response relationship between MEA and LOD duration at two years of follow-up [17]. The study by Lee and colleagues reported an increased mean LOD among LBP cases prescribed with opioid prescriptions within 2 days of the initial ED visit by 3.8 days than LBP cases who were not prescribed opioids, but the relationship between EOP and LOD was not statically significant [30]. This contradictory finding could be explained by the differences in the definition of EOP in the included studies. Lee and colleagues define EOP as opioid use within two days of the initial visit to ED, while other studies include EOP within one week [36] or two weeks of acute LBP onset [15,17,37,38].

The exact causal mechanisms explaining the relationship between EOP and LOD in acute uncomplicated LBP are not clear. Previous studies showed that EOP for acute LBP was associated with subsequent opioid dose escalation and prolonged opioid use [17,30,39] and increased healthcare utilization, including surgery [17]. Therefore, increased LOD and delayed returns to work among LBP receiving opioids could be explained by adverse side effects associated with opioids, such as drowsiness, cognitive impairment, mood disorders, falls, fractures, and other injuries including road traffic accidents [23,40,41].

The present review showed that EOP is associated with increased LOD among patients presenting with acute LBP, which supports clinical guidelines discouraging the routine use of opioids in the management of acute LBP [26,27,28]. More research is needed to identify factors influencing non-evidence-based EOP for acute LBP cases, including patient, clinician, and contextual factors. Such information may shed light on optimal strategies for bridging the gap between evidence and practice relating to EOP for LBP.

### Strengths and Limitations

The present study used a comprehensive search strategy covering relevant bibliographic databases. In addition, included studies had large longitudinal samples of LBP cases extracted from WC administrative databases capturing detailed information on medical diagnoses, medical care, and wage replacement for work disability provided for cases presenting with acute LBP, with follow-up durations ranging between one and two years. In addition, all included studies except one were of good methodological quality.

One of the limitations of this systematic review is that we were unable to quantitatively synthesize the data using meta-analysis due to the methodological heterogeneity between the included studies. Furthermore, five of the six included studies were conducted in the US, and one study was conducted in Canada, which may reduce the generalizability of the results to other parts of the world with different healthcare systems. The WC administrative databases, the primary source of data in the included studies, lack information on several factors associated with LOD in patients with acute LBP, such as pain severity, functional limitations, psychosocial factors, occupation, job physical requirements, recovery expectations, fear-avoidance, and support at work. However, a prospective cohort study from the UK reported that opioids prescribing for acute LBP cases in primary care were associated with functional disability measured using the Roland-Morris Disability Questionnaire at 6 months of follow-up, even after adjusting for anxiety, depression, fear of movement, pain self-efficacy, and coping strategies [42]. Another limitation is that estimating LOD using wage replacement data may underestimate the true LOD and work absence because the discontinuation of indemnity payments does not imply recovery and return to work. Nevertheless, this approach is frequently used in work disability studies and was found to be an equitable measure that reasonably reflects the total work loss [43]. Furthermore, other longer-term effects of EOP such as addiction and long-term disability should be considered in future studies.

## 5. Conclusions

The use of early opioid in the management of acute uncomplicated LBP is associated with prolonged disability duration. Adherence to clinical guidelines discouraging the use of opioids for acute LBP may improve functions and prevent prolonged disabilities among patients with acute LBP. Further research on factors influencing inadequate adherence to evidence-based guidelines and optimal strategies to modify such factors may improve disability outcomes among patients presenting with acute LBP.

## Figures and Tables

**Table 1:** Characteristics of included studies.

Study	Setting and Data Source	Sample Size	Population
Gasper 2021 [36]	WC administrative database: California’s Department of Industrial Relations WC Information System	59,656 cases with acute LBP from 2009 to 2018	Mean age = 41 years, (SD = 12); 66.1% men
Shraim 2019 [37], 2015 [15]	WC administrative database: a single private insurer representing approximately 10% of the U.S. private WC market	59,360 cases with acute LBP from 2002 to 2008	Mean age = 39.4 years, SD = 10.8, 69.1% men
Lee 2016 [30]	WC administrative database: a single private insurer representing approximately 10% of the U.S. private WC market	2887 cases with acute LBP from 2009 to 2011	Mean age = 41 years, 61.7% men
Gross 2009 [38]	WC administrative database: WC board of Alberta	47,784 cases from 2000 to 2005 with acute LBP	Mean age = 37 years, SD = 12, about 67.7% men
Webster 2007 [17]	WC administrative database: a single private insurer representing approximately 10% of the U.S. private WC market	8443 cases with acute LBP from 2002 to 2003	Mean age = 40.3 years, SD = 10.4, 71.8% men

LBP: low back pain; SD: Standard Deviation; WC: Workers’ Compensation.

**Table 2:** Quality assessment of studies using the Newcastle–Ottawa scale.

Study	Selection	Comparability	Outcome	Total Quality Score
Gasper 2021 [36]	****		***	7
Shraim 2019 [37]	****	**	***	9
Lee 2016 [30]	****	**	***	9
Shraim 2015 [15]	****	**	***	9
Gross 2009 [38]	****	*	***	8
Webster 2007 [17]	****	**	***	9

Number of stars = score (* = 1, ** = 2, *** = 3, **** = 4).

**Table 3:** The relationship between EOP and length of disability.

Study	Exposure	Variables Adjusted for in Multivariable Analysis	Association between EOP and LOD
Gasper 2021 [36]	Not reported	No report of adjusted variables. Descriptive statistics were used to assess the relationship between EOP and LOD.	The opioid group had a longer median LOD by 4 days when compared to the no opioid group (medians = 30 vs. 26 days).
Shraim 2019 [37]	MEA	Age, gender, tenure, industry, injury severity, early MRI, lumbar surgery, litigation status, live–work in the same state, wage replacement rate, waiting period, retroactive period, state medical fee schedule, treating provider choice, treating provider change, MRI facility rate, Wage replacement rate, State medical fee schedule, and unemployment rate.	Increase in EOP by 100 mg MEA was associated increase in mean LOD by 0.4 day (95% CI 0.3, 0.5).
Lee 2016 [30]	MEA	Adjusted for age, gender, job tenure, early MRI, and injury severity.	The EOP group had a higher mean LOD than the no EOP group (100.8 vs. 104 days). EOP was associated with an increased hazard of longer disability duration, but this was not statistically significant (hazard ratio 1.02, 95% CI 0.91, 1.13).
Shraim 2015 [15]	MEA	Age, gender, tenure, industry, injury severity, early MRI, lumbar surgery, litigation status, live-work in the same state, wage replacement rate, waiting period, retroactive period, state medical fee schedule, treating provider choice, and treating provider change.	Increase in EOP by 100 mg MEA was associated increase in LOD by 0.4 day (95% CI 0.3, 0.4).
Gross 2009 [38]	Not reported	Adjusted for age, gender, annual salary, year, number of previous claims, and injury type.	The EOP group had a higher hazard of continuation of time loss benefits by 1.94 (95% CI 1.86, 2.02).
Webster 2007 [17]	MEA	Adjusted for age, gender, job tenure, and lower back injury severity group.	As compared to subjects who did not receive EOP, subjects who received EOP had increased mean LOD days with increasing MEA dosage: 5.2 days (95% CI −14.6, 25.0) for 1–140 mg, 21.9 days (95% CI 3.2, 40.6) for 141–225 mg, 43.8 days (95% CI 23.7, 63.9) for 226–450 mg, and 69.1 (95% CI 49.3, 89.0) for 450+ mg.

CI: confidence interval; EOP: early opioid prescribing; LOD: length of disability; MEA: Morphine Equivalent Amount; MRI: Magnetic Resonance Imaging.

## Data Availability

Not applicable.

## Supplementary Materials

The following supporting information can be downloaded at: https://www.mdpi.com/article/10.3390/ijerph191912114/s1. Supplemental File S1. PRISMA 2009 Checklist. Supplemental File S2. Full search strategy. Supplemental File S3. Newcastle-Ottawa quality assessment scale for cohort studies. Reference [44] is cited in the supplementary materials.
