# Supplementary material for: The Association between Early Opioids Prescribing and the Length of Disability in Acute Lower Back Pain: A Systematic Review and Narrative Synthesis
Source: Int J Environ Res Public Health. 2022 Sep 25;19(19):12114. doi: 10.3390/ijerph191912114 (PMC9566201; doi:10.3390/ijerph191912114)
Supplement: Supplementary file 1 [file ijerph-19-12114-s001.zip › ijerph-1910947-supplementary.pdf]

International Journal of
Environmental Research and Public Health

MDPI

Supplemental File S1: PRISMA 2009 Checklist.

Section/topic	#	Checklist item	Reported on page #
**TITLE**			
Title	1	Identify the report as a systematic review, meta-analysis, or both.	1
**ABSTRACT**			
Structured summary	2	Provide a structured summary including, as applicable: background; objectives; data sources; study eligibility criteria, participants, and interventions; study appraisal and synthesis methods; results; limitations; conclusions and implications of key findings; systematic review registration number.	1
**INTRODUCTION**			
Rationale	3	Describe the rationale for the review in the context of what is already known.	1-2
Objectives	4	Provide an explicit statement of questions being addressed with reference to participants, interventions, comparisons, outcomes, and study design (PICOS).	1-2
**METHODS**			
Protocol and registration	5	Indicate if a review protocol exists, if and where it can be accessed (e.g., Web address), and, if available, provide registration information including registration number.	2
Eligibility criteria	6	Specify study characteristics (e.g., PICOS, length of follow-up) and report characteristics (e.g., years considered, language, publication status) used as criteria for eligibility, giving rationale.	3
Information sources	7	Describe all information sources (e.g., databases with dates of coverage, contact with study authors to identify additional studies) in the search and date last searched.	2-3
Search	8	Present full electronic search strategy for at least one database, including any limits used, such that it could be repeated.	2-3
Study selection	9	State the process for selecting studies (i.e., screening, eligibility, included in systematic review, and, if applicable, included in the meta-analysis).	3
Data collection process	10	Describe method of data extraction from reports (e.g., piloted forms, independently, in duplicate) and any processes for obtaining and confirming data from investigators.	3-4

Data items	11	List and define all variables for which data were sought (e.g., PICOS, funding sources) and any assumptions and simplifications made.	3-4
Risk of bias in individual studies	12	Describe methods used for assessing risk of bias of individual studies (including specification of whether this was done at the study or outcome level), and how this information is to be used in any data synthesis.	3
Summary measures	13	State the principal summary measures (e.g., risk ratio, difference in means).	NA
Synthesis of results	14	Describe the methods of handling data and combining results of studies, if done, including measures of consistency (e.g., I^2) for each meta-analysis.	NA

Section/topic	#	Checklist item	Reported on page #
Risk of bias across studies	15	Specify any assessment of risk of bias that may affect the cumulative evidence (e.g., publication bias, selective reporting within studies).	NA
Additional analyses	16	Describe methods of additional analyses (e.g., sensitivity or subgroup analyses, meta-regression), if done, indicating which were pre-specified.	NA
**RESULTS**			
Study selection	17	Give numbers of studies screened, assessed for eligibility, and included in the review, with reasons for exclusions at each stage, ideally with a flow diagram.	4-5
Study characteristics	18	For each study, present characteristics for which data were extracted (e.g., study size, PICOS, follow-up period) and provide the citations.	5-6
Risk of bias within studies	19	Present data on risk of bias of each study and, if available, any outcome level assessment (see item 12).	NA
Results of individual studies	20	For all outcomes considered (benefits or harms), present, for each study: (a) simple summary data for each intervention group (b) effect estimates and confidence intervals, ideally with a forest plot.	6-8
Synthesis of results	21	Present results of each meta-analysis done, including confidence intervals and measures of consistency.	6-8
Risk of bias across studies	22	Present results of any assessment of risk of bias across studies (see Item 15).	NA
Additional analysis	23	Give results of additional analyses, if done (e.g., sensitivity or subgroup analyses, meta-regression [see Item 16]).	NA
**DISCUSSION**			
Summary of evidence	24	Summarize the main findings including the strength of evidence for each main outcome; consider their relevance to key groups (e.g., healthcare providers, users, and policy makers).	8-9

Limitations	25	Discuss limitations at study and outcome level (e.g., risk of bias), and at review-level (e.g., incomplete retrieval of identified research, reporting bias).	9
Conclusions	26	Provide a general interpretation of the results in the context of other evidence, and implications for future research.	9
**FUNDING**			
Funding	27	Describe sources of funding for the systematic review and other support (e.g., supply of data); role of funders for the systematic review.	10

From: Moher D, Liberati A, Tetzlaff J, Altman DG, The PRISMA Group (2009). Preferred Reporting Items for Systematic Reviews and Meta-Analyses: The PRISMA Statement. PLoS Med 6(6): e1000097. doi:10.1371/journal.pmed1000097

For more information, visit: **www.prisma-statement.org**.

Supplemental File S2. Full search strategy.

	Opioid	Low Back Pain	Work Disability
**MEDLINE**	exp analgesics, opioid/<br>opioid#<br>opiate#<br>opium<br>papaver<br>buprenorphine<br>codeine<br>dextropropoxyphene<br>fentanyl<br>hydrocodone<br>hydromorphone<br>levorphanol<br>meperidine<br>methadone<br>morphine<br>oxycodone<br>oxymorphone<br>pentazocine<br>pethidine<br>propoxyphene<br>tramadol<br>sufentanil	exp low back pain/<br>exp sciatica/<br>sciatica<br>"Low back pain"<br>"Lower back pain"<br>"Low back injury"<br>"Low back injuries"<br>lumbago<br>"lumbosacral pain"<br>"lumbar pain"<br>radiculopathy<br>"radicular pain"	exp return to Work/<br>Insurance, Disability/<br>Sick leave/<br>Absenteeism/<br>work adj3 disability<br>work adj3 absence<br>work adj3 absenteeism<br>work adj3 incapacity<br>sick adj3 leave<br>sickness adj3 leave<br>sick adj3 period<br>sickness adj3 period<br>sick adj3 duration<br>sickness adj3 duration<br>disability adj3 duration<br>disability adj3 period<br>"time lost" adj3 work<br>"time loss" adj3 work<br>return# adj3 work<br>"back to" adj3 work<br>worktime adj3 loss<br>"work time" adj3 loss<br>workday# adj3 loss<br>worktime adj3 lost<br>"work time" adj3 lost<br>workday# adj3 lost
**EMBASE**	exp opiate/<br>exp narcotic agent/<br>opioid#<br>opiate#<br>opium<br>papaver<br>buprenorphine<br>codeine<br>dextropropoxyphene<br>hydromorphinone<br>fentanyl<br>hydrocodone<br>hydromorphone<br>levorphanol<br>meperidine<br>methadone<br>morphine<br>oxycodone<br>oxymorphone<br>pentazocine<br>pethidine<br>propoxyphene	exp low back pain/<br>exp ischialgia/<br>sciatica/<br>Sciatica<br>"Low back pain"<br>"Lower back pain"<br>"Low back injury"<br>"Low back injuries"<br>lumbago<br>"lumbosacral pain"<br>"lumbar pain"<br>radiculopathy<br>"radicular pain"	work disability/<br>work resumption/<br>return to work/<br>absenteeism/<br>work adj3 disability<br>work adj3 absence<br>work adj3 absenteeism<br>work adj3 incapacity<br>sick adj3 leave<br>sickness adj3 leave<br>sick adj3 period<br>sickness adj3 period<br>sick adj3 duration<br>sickness adj3 duration<br>disability adj3 duration<br>disability adj3 period<br>"time lost" adj3 work<br>"time loss" adj3 work<br>return# adj3 work<br>"back to" adj3 work<br>worktime adj3 loss<br>"work time" adj3 loss

	tramadol<br>sufentanil		workday# adj3 loss<br>worktime adj3 lost<br>"work time" adj3 lost<br>workday# adj3 lost
**CINAHL**	exp analgesics, Opioid/<br>exp narcotics/<br>opioid#<br>opiate#<br>opium<br>papaver<br>buprenorphine<br>codeine<br>dextropropoxyphene<br>hydromorphinone<br>fentanyl<br>hydrocodone<br>hydromorphone<br>levorphanol<br>meperidine<br>methadone<br>morphine<br>oxycodone<br>oxymorphone<br>pentazocine<br>pethidine<br>propoxyphene<br>tramadol<br>sufentanil	exp Low Back Pain/<br>sciatica/<br>Sciatica<br>"Low back pain"<br>"Lower back pain"<br>"Low back injury"<br>"Low back injuries"<br>lumbago<br>"lumbosacral pain"<br>"lumbar pain"<br>radiculopathy<br>"radicular pain"	Insurance, Disability/<br>Absenteeism/<br>Sick Leave/<br>Employee, Disabled/<br>work adj3 disability<br>work adj3 absence<br>work adj3 absenteeism<br>work adj3 incapacity<br>sick adj3 leave<br>sickness adj3 leave<br>sick adj3 period<br>sickness adj3 period<br>sick adj3 duration<br>sickness adj3 duration<br>disability adj3 duration<br>disability adj3 period<br>"time lost" adj3 work<br>"time loss" adj3 work<br>return# adj3 work<br>"back to" adj3 work<br>worktime adj3 loss<br>"work time" adj3 loss<br>workday# adj3 loss<br>worktime adj3 lost<br>"work time" adj3 lost<br>workday# adj3 lost

Supplemental File S3. Newcastle-Ottawa quality assessment scale for cohort studies.

Note: A study can be awarded a maximum of one star for each numbered item within the Selection and Outcome categories. A maximum of two stars can be given for Comparability.

Selection

(1) Representativeness of the exposed cohort
(a) truly representative of the average ________________ (describe) in the community ❋
(b) somewhat representative of the average _______________ in the community ❋
(c) selected group of users eg nurses, volunteers
(d) no description of the derivation of the cohort

(2) Selection of the non exposed cohort
(a) drawn from the same community as the exposed cohort ❋
(b) drawn from a different source
(c) no description of the derivation of the non exposed cohort

(3) Ascertainment of exposure
(a) secure record (eg surgical records) ❋
(b) structured interview ❋
(c) written self report
(d) no description

(4) Demonstration that outcome of interest was not present at start of study
(a) yes ❋
(b) no

Comparability

(1) Comparability of cohorts on the basis of the design or analysis
(a) study controls for ______________ (select the most important factor) ❋
(b) study controls for any additional factor ❋ (This criteria could be modified to indicate specific control for a second important factor.)

Outcome

(1) Assessment of outcome
(a) independent blind assessment ❋
(b) record linkage ❋
(c) self report
(d) no description

(2) Was follow-up long enough for outcomes to occur
(a) yes (select an adequate follow up period for outcome of interest) ❋
(b) no

(3) Adequacy of follow up of cohorts
(a) complete follow up - all subjects accounted for ❋
(b) subjects lost to follow up unlikely to introduce bias - small number lost - > ____ % (select an adequate %) follow up, or description provided of those lost) ❋
(c) follow up rate < ____% (select an adequate %) and no description of those lost
(d) no statement
